# Catalyst characteristics of the composite catalyst of Ru–Sn and Pd for hydrogenation of terephthalic acid

**DOI:** 10.1039/d3ra04327d

**Published:** 2023-09-08

**Authors:** Zhang Ruijie, Jin Haibo, Ma Lei, Yang Suohe

**Affiliations:** a School of New Materials and Chemical Engineering, Beijing Institute of Petrochemical Technology/Beijing Key Laboratory of Fuel Cleaning and Efficient Catalytic Emission Reduction Technology Beijing 102617 P.R. China jinhaibo@bipt.edu.cn

## Abstract

1,4-Cyclohexanedimethanol (CHDM) is a premium polyester monomer. In this paper, a series of Ru– Sn/γ-Al_2_O_3_ and Pd/γ-Al_2_O_3_ bimetallic composite catalysts were prepared by the impregnation method. The effects of preparation conditions such as active components, preparation methods, and the ratio of Sn to Ru on the reaction activity of the catalysts were studied. The bimetallic composite catalysts show desirable properties including high surface area and high dispersion of active centers. Pd-based catalysts were found to be effective in the initial stage of benzene ring hydrogenation, while Ru-based catalysts were found effective in the subsequent stage of carboxyl group hydrogenation. The Ru–Sn bimetallic active center was predominantly located in the Ru-based catalysts. In addition, the effects of reaction parameters such as reaction temperature, pressure, and time on hydrogenation reactions were also examined. It was found that increasing reaction temperature had no significant effect on the conversion rate of PTA, but it did affect the yield of CHDM, initially increasing and then decreasing. Increasing the reaction pressure resulted in a gradual increase in the yield of CHDM, while the conversion rate of PTA remained unchanged. The composite catalyst with a Sn/Ru ratio of 0.5 demonstrated the best performance, achieving a CHDM conversion of 96.3% and a yield of 72.2%.

## Abstract 2

In this paper, two-stage catalysts are creatively and physically mixed, and two-step hydrogenation is combined into one step.

## Graphical abstract

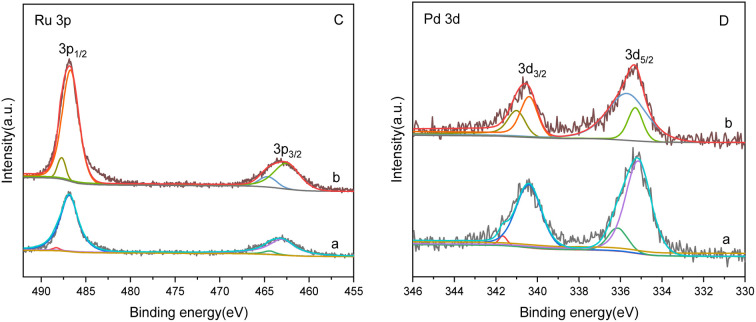

## Introduction

1,4-Cyclohexanedimethanol (CHDM) is widely used as a substitute for ethylene glycol monomer in the production of various polyester products, such as polyester fiber, polyester electrical appliances, unsaturated polyester resin, polyester glaze, polyurethane foam, hydraulic oil, and lubricant production.^1–3^ However, the raw materials for CHDM production primarily come from the traditional petrochemical industry, which has limited oil reserves. So it is necessary to find alternative raw materials. Purified terephthalic acid (PTA) has been identified as a potential raw material for CHDM production due to its low cost, environmental friendliness, and simple preparation process.

Catalysts based on precious metals and transition metals, such as ruthenium, rhodium, platinum, and nickel, have been widely employed for benzene ring hydrogenation under mild conditions.^4^ Copper-based catalysts have traditionally been used for the hydrogenation of esters and carboxylic acids to produce alcohol. In the process of CHDM production *via* two-step hydrogenation of PTA, the conversion rate of PTA and the yield of CHDM are relatively high, suggesting that PTA is an excellent raw material for CHDM synthesis. At present, Ru–Sn bimetallic catalyst is commonly used to convert PTA into CHDM. However, the reaction pressure requires high pressures, typically above 10 MPa, resulting in high energy consumption and operational risks. Therefore, it is necessary to improve the Ru–Sn bimetallic catalyst to enable the conversion of PTA into CHDM at lower pressure.

In the recent ten years, the direct hydrogenation of PTA to produce CHDM has undergone significant advancements. The PTA hydrogenation process can be divided into one-step and two-step hydrogenation methods. Zhang *et al.*^5^ presented a two-step hydrogenation process using PTA as raw material to prepare CHDM. In the first step, a 5% Pd/C catalyst was employed at a reaction temperature of 250 °C, a hydrogen pressure of 12 MPa, and a reaction time of 1 h. The conversion, yield, and selectivity of CHDA were 99%, 98%, and 99% respectively. In the second step, a 5% Ru–5% Sn/C catalyst was used at a reaction temperature of 250 °C, a hydrogen pressure of 10 MPa, and a reaction time of 6 h. The results showed the conversion of CHDA of 99.4%, the yield of CHDM of 97.7%, and the selectivity of CHDM of 98.3%. However, these two steps involve different reaction temperatures and pressures, leading to the complexity of operation and production costs. This complexity can result in product loss during the operation, and intermediate products are challenging to separate the intermediate products for further hydrogenation.

In recent years, a one-step liquid-phase hydrogenation method for preparing CHDM has been proposed. A U.S. patent discloses a liquid phase one-step method for preparing CHDM using 5.0% Ru, 3.5% Sn, and 5.6% Re/C. Under conditions of 250 °C and 15 MPa for 5 h, the conversion of PTA and the yield of CHDM reached 97% and 42%, respectively. Zhao^6^ used terephthalic acid as raw material and an Sn–Ru–B/Al_2_O_3_ catalyst. The temperature and pressure in the first stage were 150 °C and 6 MPa, respectively, while in the second stage, the temperature and pressure were 230 °C and 10 MPa, respectively. The conversion of terephthalic acid was 100%, and the yield of CHDM was 86.32%. Choi *et al.*^7^ employed a composite metal catalyst to prepare CHDM using terephthalic acid as raw material. The first metal catalyst is 2.5% Pd/Y-zeolite, and the second metal catalyst is 2.5% Ru-2.5% Sn-1.5% Pt/Y-zeolite. The two catalysts were placed in a fixed bed reactor, and the reaction was conducted at a temperature of 230 °C, with a reaction pressure of 8 MPa. The conversion of PTA was 100%, and the selectivity of CHDM was 93%. The composite metal catalyst simplified the experimental setup, reduced the reaction operation process, and improved the reaction efficiency, it had a high content of noble metal, intricate preparation requirements, and high cost. Therefore, further research is needed to explore catalysts for the one-step hydrogenation of PTA to CHDM.

CHDM exists in two isomeric forms: *cis*-isomer and *trans*-isomer. The *trans*-isomer structure has a symmetrical lattice structure, high lattice energy, and exhibits properties such as a high melting point and excellent heat resistance. Consequently, it is used to produce polyester products with high heat resistance and superior performance.^8,9^

In summary, the production of CHDM from PTA *via* hydrogenation has gained significant attention. In summary, the production of CHDM from PTA *via* hydrogenation has gained significant attention. The utilization of Ru–Sn/γ-Al_2_O_3_ and Pd/γ-Al_2_O_3_ bimetallic composite catalysts for the hydrogenation of benzene rings and carboxyl groups in the synthesis of CHDM has shown promising results. However, there is a need to investigate various factors, such as active components, preparation methods, and the ratio of Sn to Ru, to optimize the catalytic activity of the composite catalysts. Moreover, the reaction conditions, including temperature, pressure, and time, also play a crucial role in the hydrogenation reaction. By studying and optimizing these parameters, the conversion rate of PTA and the yield of CHDM can be improved. Additionally, it is essential to consider the isomeric structure of CHDM and its impact on the properties of polyester products.

**Fig. 1:**
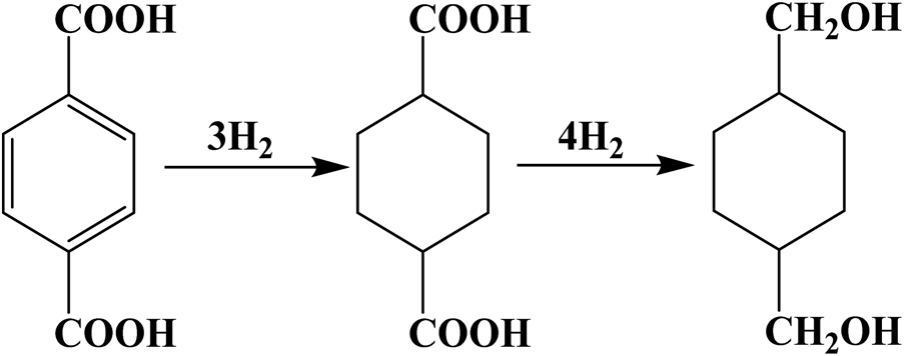
Reaction paths of the PTA hydrogenation.

The Ru–Sn supported catalyst, known for its hydrogenation of esters and carboxylic acids, demonstrates good performance. The hydrogenation of the phenyl and carboxyl groups of PTA in the reactor is facilitated by multifunctional catalysts with different active centers. And the reaction pathway is shown in Fig. 1. Sn can activate carboxyl groups of esters and carboxylic acids, such as Lewis acids, and improves the selectivity of alcohol. Ruthenium, on the other hand, facilitates the activation of hydrogen and the hydrogenation of phenyl groups.

Precious metals are commonly utilized as active components in catalysts due to their low loading and good catalytic activity at low temperatures. Compared with other precious metals, palladium is widely used as an active component because of its superior thermal stability, excellent catalytic performance, and strong activation ability for H_2_ molecules.^10,11^ Although Ru and Pd are immiscible in the atomic state, there is a slight transfer of the electrons from the Pd atom to the Ru atom, which leads to the enhancement of catalytic activity.^12^

In this study, Ru–Sn/γ-Al_2_O_3_ and Pd/γ-Al_2_O_3_ bimetallic composite catalysts were prepared from terephthalic acid using the impregnation method the one-step hydrogenation of PTA was conducted to produce CHDM was prepared from by the effects of reaction parameters such as temperature, pressure, and time on hydrogenation reactions were investigated. In addition, the supported catalyst was characterized by temperature-programmed reduction, X-ray photoelectron spectroscopy, X-ray diffraction, and scanning electron microscope. These characterizations provide insights into the structure and composition of the catalysts, allowing for a better understanding of their catalytic properties.

## Experimental

### Materials

The following materials were used in the experiment: terephthalic acid (≥99%, MERYER (Shanghai) CHEMICAL Technology Co., Ltd.); 1,4-cyclohexanedimethanol (≥99%, MERYER (Shanghai) CHEMICAL Technology Co., Ltd.); 1,4-cyclohexanedicarboxylic acid (≥98, MERYER (Shanghai) CHEMICAL Technology Co., Ltd.); γ-Al_2_O_3_ (China National Pharmaceutical Group Corporation); methanol (≥99.5%, MERYER (Shanghai) CHEMICAL Technology Co., Ltd.); sodium borohydride (≥98%, China National Pharmaceutical Group Corporation); glycerol (≥99%, China National Pharmaceutical Group Corporation); PdCl_2_ (≥99%, Tianjin Guangfu Institute of Fine Chemical Industry); RuCl_3_·3H_2_O (≥98%%, Energy Chemical); SnCl_2_·2H_2_O (≥98%%, MERYER (Shanghai) CHEMICAL Technology Co., Ltd.).

### Catalysts preparation

The Ru–Sn/γ-Al_2_O_3_ catalyst: a certain amount of SnCl_2_·2H_2_O was dissolved in deionized water in a beaker, and a certain amount of pretreated γ-Al_2_O_3_ carrier was added. The mixture was soaked for 10 h, and then the impregnated samples were dried in a vacuum drying oven at 80 °C for 2 h. Subsequently, the samples were calcined in air at 500 °C for 2 h. A certain amount of RuCl_3_·3H_2_O was dissolved in deionized water in a beaker, and the calcined sample was added and soaked for 10 h at room temperature. Then the excess NaBH_4_ was dissolved in NaOH solution with a pH value of 9 to reduce the catalyst sample. After reduction, it was washed with deionized water many times until the pH value reached 7. Finally, it was dried in air at 80 °C for 10 h, and the Ru–Sn/γ-Al_2_O_3_ catalyst was obtained. In addition, the Pd/γ-Al_2_O_3_ catalyst was prepared by the same method.

### Catalysts characterization

The reduction performance and hydrogen absorption performance of the catalyst were studied using the Auto Chem II 2920 temperature programmed reduction instrument (TPR). The temperature of the catalyst was raised from 40 °C to 800 °C at a rate of 10 °C min^−1^ with a hydrogen–argon mixture containing 5% hydrogen.

The XRD spectrum of the catalyst was analyzed using the D8 Venture X-ray powder diffractometer. The range of 2*θ* angle was 20° to 80° at a scanning rate of 10°/min. The morphology of the catalyst was observed using a Zeiss G300 field emission scanning electron microscope. XPS was measured and analyzed using the Thermo Fisher Scientific EscaLab 250 Xi recorder and the energy of the AI K alpha radiation source was 1486.8 eV. The specific surface area of the catalyst was analyzed using the BELSORP-mini II specific surface area and porosity analyzer. The catalyst was tested by the BELCAT-B CO chemisorption in Bayer, Japan. The sample was purged and reduced for 1 h at 200 °C in the atmosphere of 10% H_2_/Ar mixed gas. When the catalyst was cooled to room temperature, it was purged with pure He gas at a flow rate of 25 mL min^−1^ until the baseline was stable, and then CO was introduced for pulse adsorption.

### Experimental instruments

In this experiment, the high-pressure reactor (160 mL) from Parr Instruments Company of the United States was used as the performance evaluation device for catalyst hydrogenation. During the reaction, 3.0 g of PTA as substrate and 0.6 g of Pd/γ-Al_2_O_3_, and 1.0 g of Ru–Sn/γ-Al_2_O_3_ catalyst were added into the reactor. The reactor was purged with N_2_ three times, and then replace with H_2_ three times. The reactor was pressurized with H_2_ to a pressure of 4 MPa. Subsequently, the reactor was heated to 180 °C and the pressure at this temperature was 5 MPa. After 1.5 h of reaction, the temperature was raised to 230 °C, the pressure was increased to 8 MPa, and the reaction continued for 5 h with pressure replenishment every half hour. After the reaction, the reaction solution was filtered to remove the solid catalyst, and the collected catalyst was washed with ethanol to remove the surface residue. The catalyst was dried at 120 °C for 10 h. The target product was obtained by rotary evaporation of the reaction solution, and the resulting product was dissolved in a certain amount of methanol for analysis using gas chromatography.

Liquid samples were analyzed using gas chromatography (GC-2014) equipped with an HP-5 chromatographic column and a flame ionization detector (FID). The initial box temperature was set at 140 °C and kept for 2 minutes, then heated to 200 °C at the rate of 12 °C min^−1^, and maintained at 200 °C for 2 minutes. The FID temperature was set to 295 °C and the inlet temperature was set to 290 °C. The product was quantitatively analyzed using the internal standard method. The conversion and yield of the product were calculated using the following eqn (1) and (2):1
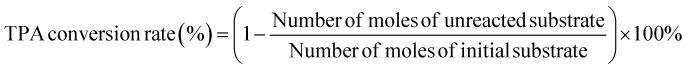
2
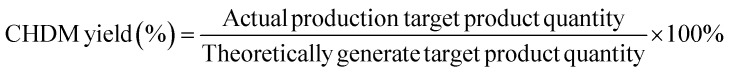


## Results and discussion

Using PTA as raw material, CHDM was prepared by a two-step hydrogenation process. The first step involved the hydrogenation of the benzene ring of terephthalic acid to generate CHDA, and the second step involved the hydrogenation of the carboxyl group to obtain the target product CHDM. The benzene ring hydrogenation in the first step is easier than the carboxyl hydrogenation in the second step, and the reaction conditions are milder. As a diol, CHDM may undergo hydrogen hydrolysis, polymerization, and chain scission under high temperature and high pressure.^13,14^ Therefore, the conversion of PTA not only depends on the hydrogenation activity of the catalyst but also the occurrence of side reactions. To develop efficient catalysts, a series of supported catalysts were prepared using the impregnation method, and the effect of different catalyst compositions on the hydrogenation performance of PTA was investigated.

### Influence of different active components on catalyst activity

**Fig. 2:**
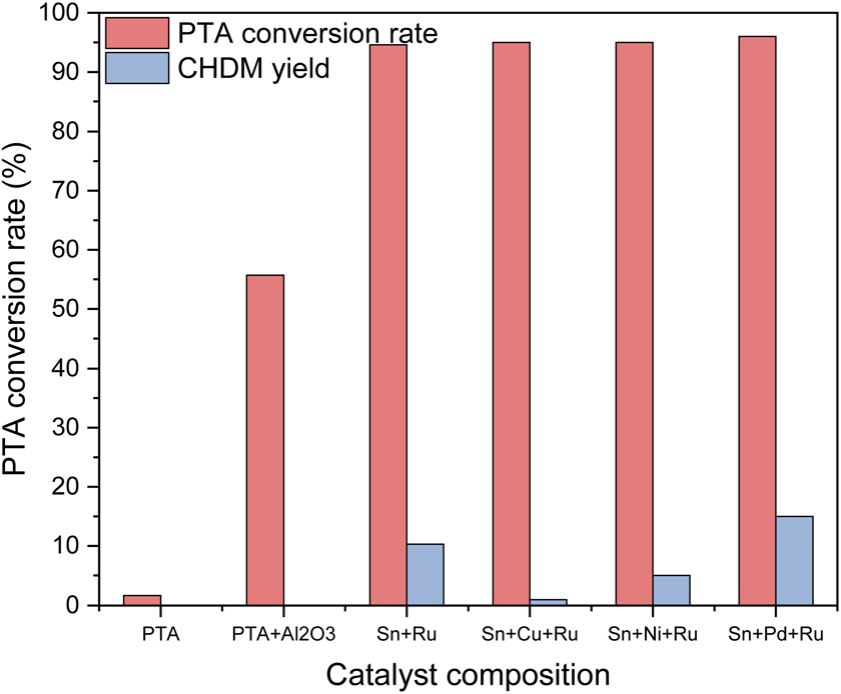
Effect of loading different metals on hydrogenation reaction.

Various catalysts containing different active components were used to selectively hydrogenate PTA to CHDM, aiming to determine the optimal metal composition of the catalyst. The catalytic results are shown in Fig. 2. It can be seen that in the absence of any loaded metals, only raw materials and blank carriers are added, resulting in no production of target products. When additional metals such as Cu, Ni, Pd, and other third metals were loaded on the Ru–Sn catalyst,^15^ respectively, the conversion rate of PTA exceeded 95%, while the yield of CHDM depended on the metal composition of the catalyst. The results indicate the addition of a Pd catalyst exhibited the best performance. Furthermore, loading Pd on a blank carrier alone and using it in combination with the Ru–Sn catalyst yielded favorable results. These findings suggest that the synergistic effect of the three metal elements in the catalyst positively influences the selective hydrogenation of PTA to CHDM.

Ru catalyst is primarily active in the hydrogenation of C=C bonds. However, the addition of a small amount of Sn can activate the hydrogenation of C=C and C=O bonds, leading to a significant enhancement in the hydrogenation activity of C=O. The sharp increase in C=O hydrogenation activity on the Ru–Sn catalyst may be attributed to the increased electron density of Ru, which facilitates the adsorption and hydrogenation of C=O on the catalyst surface. Therefore, C=O hydrogenation and C=C hydrogenation can be selectively controlled by adding additive Sn to adjust and improve the catalytic performance of the Ru catalyst, to realize the high selectivity of CHDM of the Ru catalyst in hydrogenation reaction.^16^ The combination of Ru–Sn catalyst enables the conversion of PTA into CHDM, with Ru serving as the primary active component. The addition of Sn accelerates the polarization of the C=O bond in the CHDA molecule,^17^ making the formation of CHDM easier. The poor performance of Sn–Cu–Ru and Sn–Ni–Ru catalysts can be attributed to the increased generation of by-products after the addition of Cu and Ni, resulting in a decrease in the yield of CHDM. Pd-based catalysts exhibit higher activity in the hydrogenation of C=C double bonds compared to other precious metal catalysts.^18,19^ This can be attributed to the favorable electronic configuration of Pd metal with its 4d^10^, and it is easier to chemically adsorb with H_2_ because of the holes in its d-orbital electrons. As a result, Pd exhibits strong adsorption–desorption performance of H_2_, promoting the catalytic hydrogenation of the benzene ring. At the same time, the strong metal-carrier interaction between Pd and γ-Al_2_O_3_ ensures stable catalytic activity on particle size, indicating a positive correlation between the rate of selective hydrogenation of C=C and the active site of Pd.^20^ However, the effect of a Pd-based catalyst alone on carboxyl hydrogenation is not significant. In a study by Zhao *et al.*, it was found that the adsorption heat of H_2_ on the Pd–Ru catalyst is the highest, indicating the formation of a new surface electronic structure and chemical properties resulting from the interaction between Pd and Ru atoms. This strong interaction enables the catalyst to adsorb raw materials and H_2_ strongly, and show high activity and selectivity.^21^

### Effects of different preparation methods on catalyst activity

**Fig. 3:**
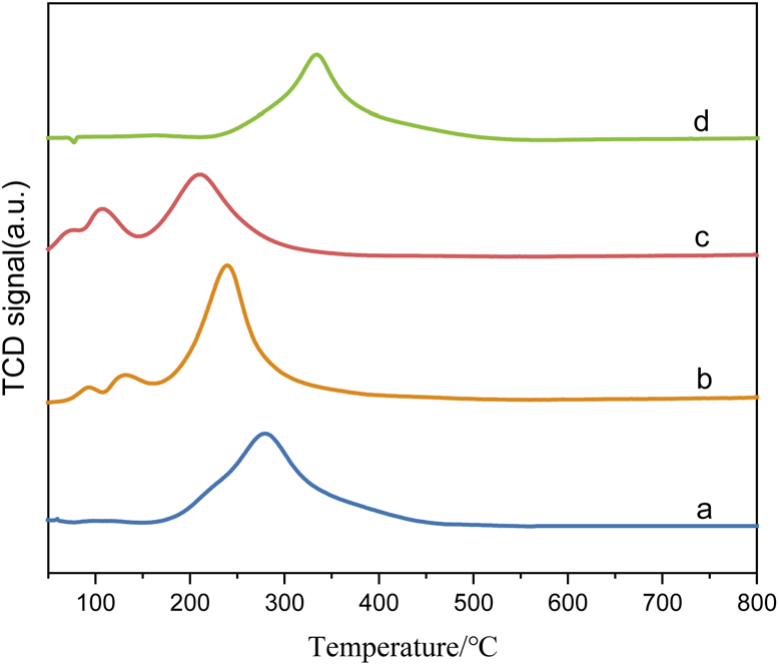
H_2_-TPR spectra of catalysts: (a) Ru–Sn–Pd/γ-Al_2_O_3_; (b) Ru–Sn/γ-Al_2_O_3_, Pd/γ-Al_2_O_3_; (c) Ru–Sn/γ-Al_2_O_3_; (d) Pd/γ-Al_2_O_3_.

Fig. 3 shows the H_2_-TPR spectra of catalysts prepared by different methods. In Fig. 3, (d) is the reduction peak of Pd, and (c) is the reduction peak of Ru–Sn. The spectra provide insights into the reduction behavior of the catalysts and the interactions between the different metals. It can be observed that the Ru–Sn–Pd/γ-Al_2_O_3_ catalyst has a single hydrogen consumption peak at 283 °C, which indicates that there is an interaction among three metals in the three-metal catalyst supported together. The interaction between these metals can influence the reduction behavior and catalytic activity of the catalyst.

On the other hand, Ru–Sn/γ-Al_2_O_3_ and Pd/γ-Al_2_O_3_ catalysts exhibit two smaller hydrogen consumption peaks at 92 °C and 132 °C, which are attributed to the reduction peaks of ruthenium chloride and ruthenium oxychloride.^22,23^ Additionally, a larger hydrogen consumption peak is observed at 243 °C, which belongs to the reduction peak of tin, ruthenium, and palladium interaction. It can be observed that the temperature of the reduction peaks loaded separately is lower than that of the reduction loaded together.

Table 1 shows the results of CO pulse adsorption on Pd/γ-Al_2_O_3_, Ru–Sn/γ-Al_2_O_3_ composite catalyst, and Ru–Sn–Pd/γ-Al_2_O_3_ catalyst. It is found that when the noble metals are supported separately, they exhibit higher dispersion, larger specific surface area, and smaller particle diameters. This observation suggests that the better dispersion of active metals on the carrier leads to the higher activity of the catalyst, which is consistent with the experimental results.

**Table 1:** CO pulse adsorption test results

Catalyst	Metal dispersion (%)	Metal surface area (m^2^ g^−1^)	Average particle diameter (nm)
Pd	1.3	5.6	89.4
Pd, Ru–Sn	16.6	62.8	7.8
Ru–Sn–Pd	13.7	52.7	9.3

**Fig. 4:**
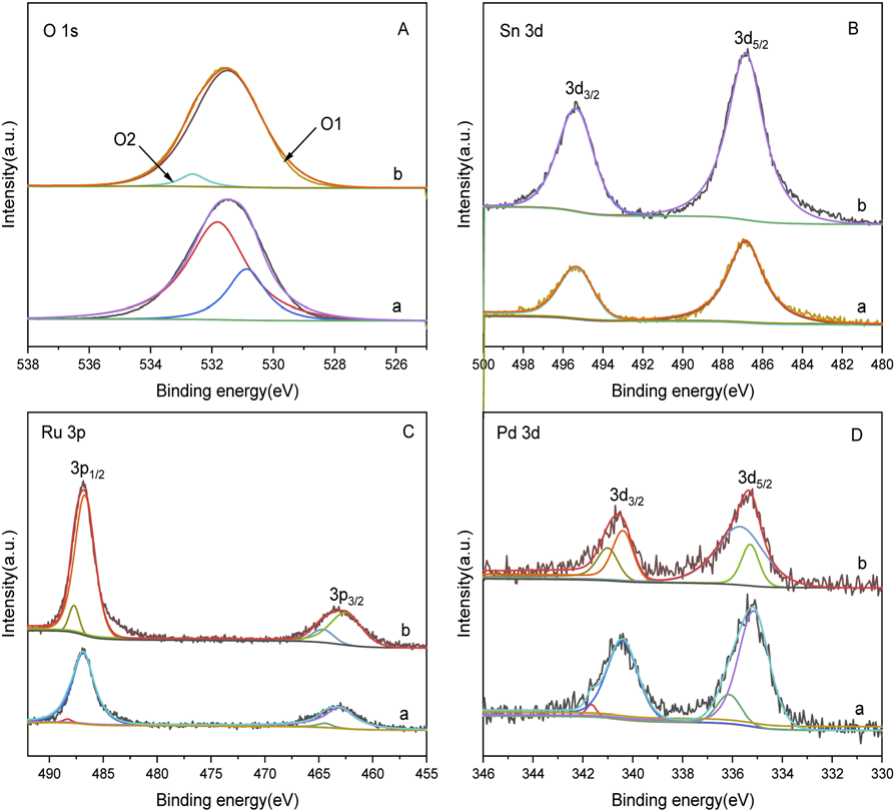
XPS spectra of catalysts with different loading methods: (A) O 1s; (B) Sn 3d; (C) Ru 3p; (D) Pd 3d. (a) Ru–Sn/γ-Al_2_O_3_, Pd/γ-Al_2_O_3_; (b) Ru–Sn–Pd/γ-Al_2_O_3_.

The XPS spectrum in Fig. 4 provides insights into the surface composition and electronic states of the catalysts with different loading modes. Fig. 4(a) represents the catalyst with ruthenium and palladium loaded separately. After mixing, the content of ruthenium in the catalyst is 3.7 wt%, the molar ratio of tin to ruthenium is 0.5 : 1, and the content of palladium is 0.07 wt%. Fig. 4(b) represents the catalyst supported by ruthenium and palladium together, in which the content of ruthenium is 6 wt%, the atomic ratio of tin to ruthenium is 0.5 : 1, and the content of palladium is 2 wt%, and the peak intensity in XPS is in direct proportion to the substance content. It can be observed that the peak intensities of Sn and Ru in the separately supported catalyst are lower compared to the catalysts supported together, while the peak intensities of Pd have minimal differences.

The O 1s spectrum displays peaks at O1 and O2, corresponding to lattice oxygen and oxygen vacancies on the catalyst surface, respectively. In the separately supported catalysts, the proportion of oxygen vacancies is higher compared to the catalysts supported together. Oxygen vacancies play a crucial role in regulating the electronic structure of metal oxides, exposing more active sites, and facilitating the adsorption of H_2_ molecules and the hydrogenation reaction.

The peaks at O1 and O2 in the O 1s spectrum belong to lattice oxygen and oxygen vacancies on the catalyst surface,^24,25^ respectively. It can be found that the proportion of oxygen vacancy in separately supported catalysts is higher compared to the catalysts supported together. The oxygen vacancies on the catalyst surface play a crucial role in regulating the electronic structure of metal oxides, exposing more active sites, and facilitating the adsorption of H_2_ molecules^26^ and the hydrogenation reaction. The two peaks at 487.5 eV and 464.5 eV in the Ru 3p spectrum are characteristic peaks of Ru^δ+^, those at 486.5 eV and 462.5 eV are characteristic peaks of Ru^0^, and those at 336.4 eV and 341.3 eV in Pd 3d spectrum are characteristic peaks of Pd^2+^ and those at 335.2 eV and 340.3 eV are characteristic peaks of Pd^0^.^27^ It is observed that the proportion of Ru^0^ and Pd^0^ is higher in the separately supported catalyst, which indicates that the separately supported Pd and Ru exist as active components in their elemental form. This indicates that the separately supported Pd and Ru catalysts are suitable for this study.

**Fig. 5:**
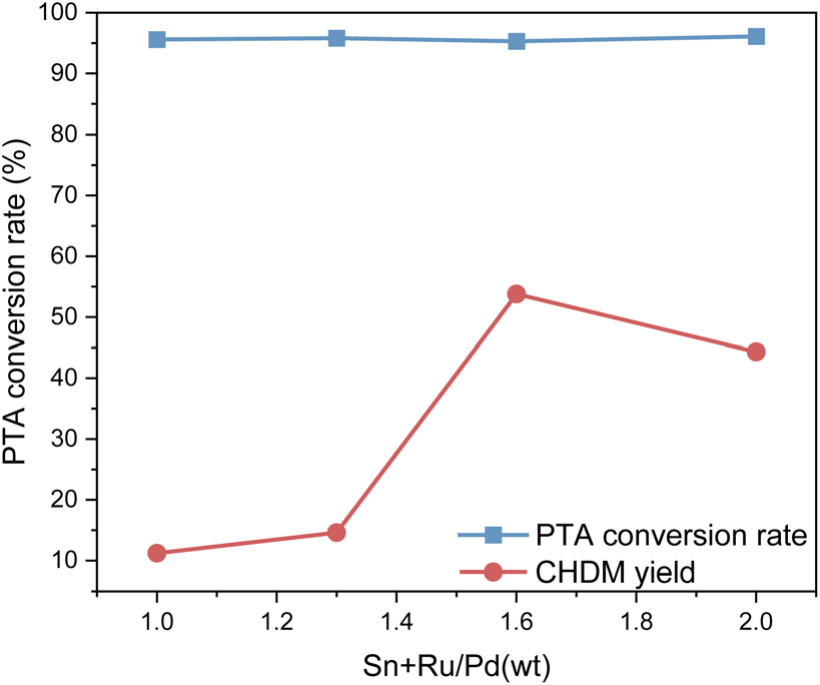
Effect of different catalyst ratios on hydrogenation reaction.

Fig. 5 shows the impact of the different proportions of the two catalysts on PTA conversion and CHDM yield. From Fig. 5, the different proportions of composite catalysts have little difference in PTA conversion, but it does affect the yield of CHDM. The results suggest that there is a synergistic effect between different metals in the composite catalysts. When the ruthenium-based catalyst is constant, the amount of palladium catalyst is not as much as possible. According to the reaction results, the proportions of the composite catalyst components to achieve the desired catalytic performance and maximize the yield of CHDM in the hydrogenation of PTA when the mass ratio of Ru–Sn/Pd is 1.6 : 1. At this time, when the Ru–Sn catalyst is 1 g, the Pd catalyst is 0.6 g.

### Effect of different Ru–Sn ratios on catalyst activity

**Fig. 6:**
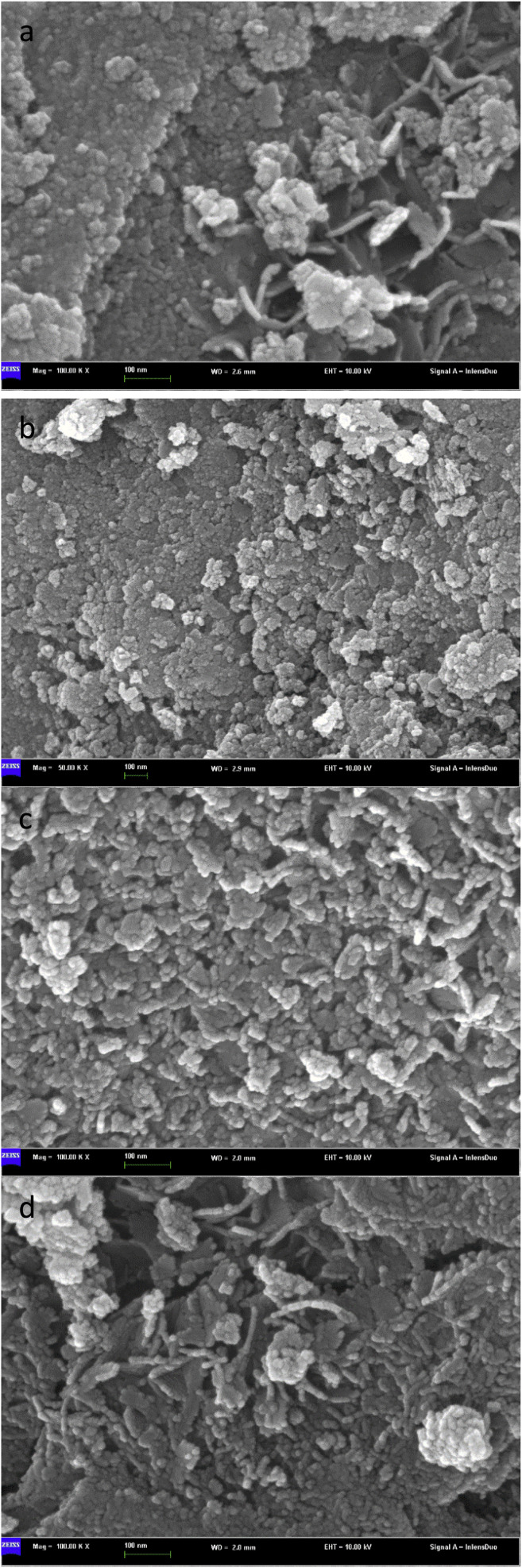
Scanning electron microscope pictures of catalysts with different Sn–Ru ratios: (a) 6% Ru/γ-Al_2_O_3_; (b) Sn/Ru : 0.2; (c) Sn/Ru : 0.5; (d) Sn/Ru : 1.1.

Fig. 6 is an SEM image of bimetallic catalysts with different ratios of Sn to Ru. From the figure, we can see the surface morphology of the catalyst. In Fig. 6(a), the SEM image represents the catalyst only loaded with Ru, revealing the accumulation of Ru I on the carrier in the form of large particles with uneven distribution. On the other hand, in Fig. 6(b)–(d), the SEM images show the catalysts after the addition of Sn, where the flocculent aggregates formed by Ru and Sn are uniformly distributed on the surface of the carrier.

The experimental results show that when Sn/Ru = 0.5, the yield of CHDM reaches the maximum. However, with the increase of tin loading, excessive Sn tends to cover the surface of Ru, leading to a reduction in the catalytic activity of the catalyst. These results are consistent with the experimental results.

**Fig. 7:**
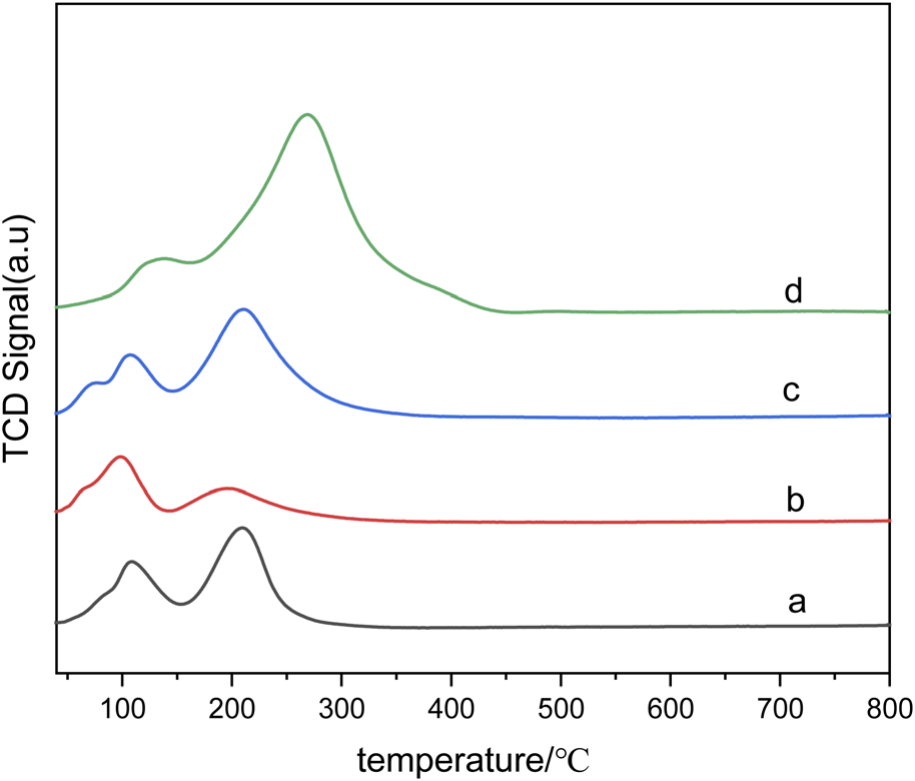
H_2_-TPR diagram of catalysts with different Sn–Ru ratios: (a) 6% Ru/γ-Al_2_O_3_; (b) Sn/Ru : 0.2; (c) Sn/Ru : 0.5; (d) Sn/Ru : 1.1.

Fig. 7 presents the H_2_-TPR diagram of bimetallic catalysts with different Sn–Ru ratios. In Fig. 7(a), the H_2_-TPR diagram corresponds to catalysts only loaded with Ru, and the reduction peaks of ruthenium chloride and ruthenium oxychloride correspond to those of monometallic Ru catalysts at 108 °C and 208 °C, respectively. Because chlorine is not completely removed in the steps of calcination and washing, RuCl_3_ and other ruthenium chloride compounds which are not easily reduced exist in the catalyst. As can be seen from Fig. 7, with the addition of Sn, the reduction peak of RuCl_*x*_ shifts to the high-temperature region. This shift suggests that the addition of Sn changes the interaction between alumina and ruthenium. In addition, with the increase of Sn loading, the hydrogen consumption also increases gradually, and the highest temperature of the reduction peak increases with the increase of Sn/Ru ratio. The peak observed in the figure is attributed to the reduction peak of Ru–Sn interaction, indicating the formation of Ru–Sn alloy in Ru–Sn bimetallic catalyst.

**Fig. 8:**
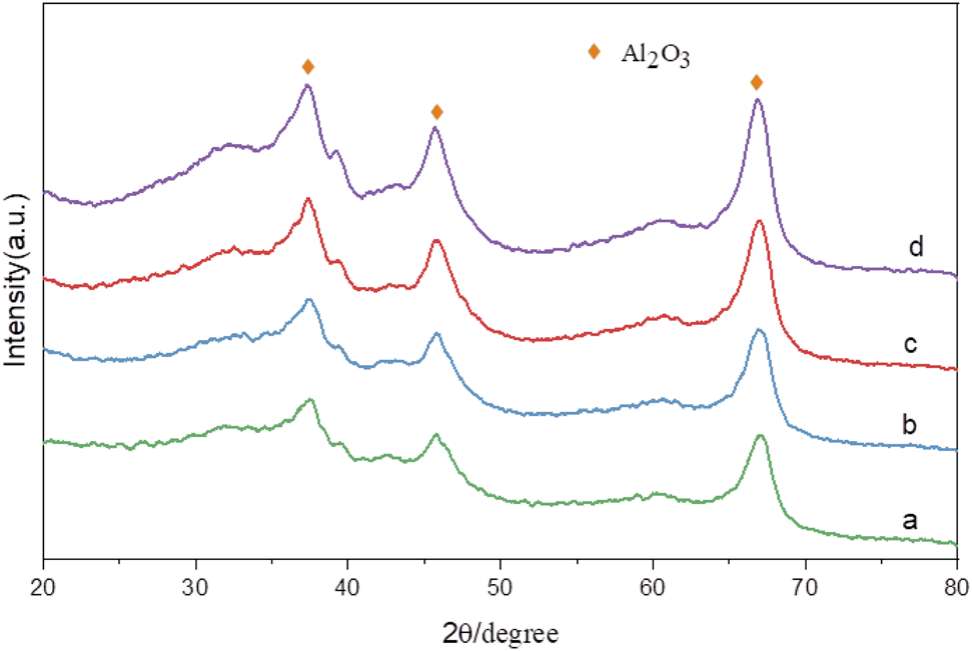
XRD patterns of catalysts: (a) γ-Al_2_O_3_; (b) 2% Ru–Sn/γ-Al_2_O_3_; (c) 6% Ru–Sn/γ-Al_2_O_3_; (d) 10% Ru–Sn/γ-Al_2_O_3_.

Fig. 8 shows the X-ray diffraction spectra of catalysts with different Ru contents at the same Sn–Ru ratio. It can be seen from the XRD pattern that the four catalysts have obvious characteristic diffraction peaks at 37.60°, 45.79°, and 66.76°, and these three characteristic diffraction peaks correspond to the three crystal faces of γ-Al_2_O_3_ (311), (400) and (440) respectively. The characteristic peak of the Ru element was not detected in the XRD pattern, indicating that the Ru element was uniformly dispersed on the catalyst.^28^ The characteristic peak of Sn_2_Ru was observed at 42.63°, and the characteristic peak at 37.32° and 67.14° was covered by the strong diffraction peak of γ-Al_2_O_3_ support. However, it was observed that the characteristic peak intensity of Sn_2_Ru gradually increased with the increase of ruthenium load, indicating that Sn_2_Ru was formed on the catalyst. The results are consistent with the H_2_-TPR analysis.

**Fig. 9:**
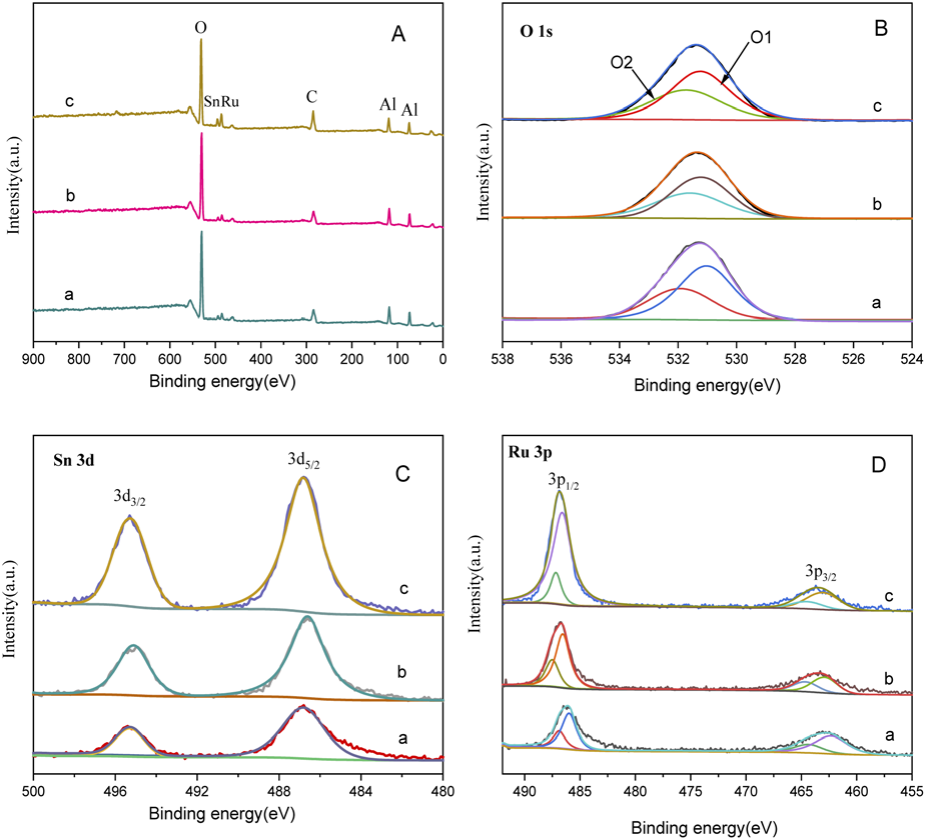
XPS spectrum of catalysts with different Sn–Ru ratios: (A) general spectrum; (B) O1s; (C) Sn 3d; (D) Ru 3p; (a) Sn/Ru: 0.2; (b) Sn/Ru: 0.5; (c) Sn/Ru: 1.1.

**Fig. 10:**
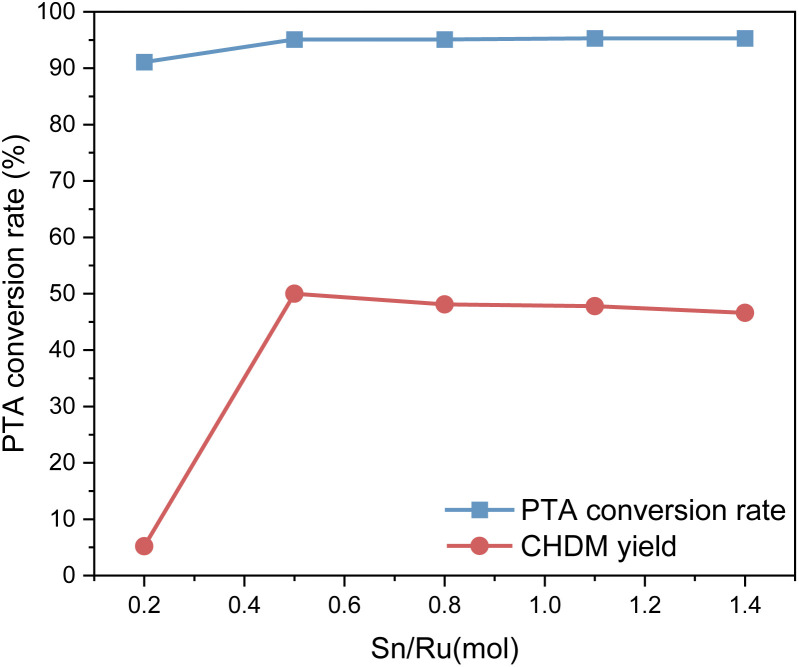
Effect of different Sn–Ru ratios on hydrogenation reaction.

Fig. 9 presents the XPS spectrum of catalysts with different Sn–Ru ratios. In Fig. 8(A), the general spectrum of catalysts with different Sn–Ru ratios is shown. It can be observed that the characteristic peak area of Sn increases gradually with the increase of Sn loading, indicating an enhanced loading effect of Sn on the catalyst. Fig. 8(B) shows the O 1s spectrum of catalysts with different Sn–Ru ratios. The proportion of oxygen defects remains relatively unchanged with increasing Sn content, indicating that the content of Sn has little influence on the existing form of oxygen in the catalyst.^29^Fig. 8(C) and (D) show the Sn 3d and Ru 3p XPS spectra of catalysts with different Sn–Ru ratios, respectively. From Fig. 8(C) and (D), it can be seen that the Sn 3d orbit splits into Sn 3d_3/2_ and Sn 3d_5/2_, and the Ru 3p orbit spins into Ru 3p_1/2_ and Ru 3p_3/2_, respectively. From the XPS spectrum of Sn 3d, it can be seen that with the increase of the ratio of Sn to Ru, the peak areas of Sn 3d_3/2_ and Sn 3d_5/2_ also increase gradually, and the binding energy of the XPS spectrum of Sn 3d at 486.9 eV corresponds to Sn^δ+^,^30,31^ which indicates that the tin in the catalyst exists in the ionic state. Ru metal on the γ-Al_2_O_3_ carrier is not completely reduced to Ru,^4,20^ so part of Ru in the catalyst exists in the Ru^δ+^ state. From the XPS diagram of Ru 3p, it can be seen that the peak area of Ru 3p_1/2_ increases with the increase of the ratio of Sn to Ru, while the peak area of Ru 3p_3/2_ is almost unchanged. The area of the peak attributed to Ru^0^ is much larger than that of RuO_*x*_, which indicates that the presence of tin increases the content of Ru^0^ on the catalyst.^32^ With the increase of the ratio of tin to ruthenium, the peak of Ru 3p shifts to the left, which indicates that Sn can make Ru in an electron-rich state, which is beneficial to the reduction of Ru elements.^33^ XPS characterization shows that there is an interaction between Sn and Ru. With the increase of Sn loading, the BE at Ru 3p_1/2_ increased from 486.0 eV to 486.9 eV, which was due to the formation of Sn_2_Ru alloy. Based on the results of XRD, H_2_-TPR, and XPS characterization of the Ru–Sn catalyst, it can be shown that after the reduction of the Ru–Sn bimetallic catalyst, the main species are Ru^0^ and Sn_2_Ru alloy. However, using Ru/γ-Al_2_O_3_ single metal catalyst, the yield of the target product CHDM is only 3%, indicating that the single ruthenium active site is not the main active site. Therefore, the most important catalytic active site is the Ru–Sn bimetallic active center (Fig. 10).

According to the data in Table 2, with the increase of the Sn/Ru ratio, the specific surface area of the catalyst showed a downward trend; with the increase of Sn loading, more and more surface sites on the catalyst were covered, which also indicated that Sn element was successfully loaded on the carrier. When the Sn/Ru ratio is 0.5, the catalyst exhibits the smallest pore volume and pore diameter. This indicates that the Sn element has effectively the pores of γ-Al_2_O_3_, leading to a decrease in pore volume and size. When the Sn/Ru ratio increases to 1.1, the specific surface area of the catalyst decreases further. However, the pore volume and pore diameter increase, indicating that the Sn element is not loaded into the pores of the carrier, but covers the surface of the carrier, reducing the active sites of the catalyst, thus reducing the catalytic activity.^34^ Considering these factors comprehensively, the optimal ratio of Sn/Ru is determined to be 0.5. This ratio ensures the successful loading of Sn into the catalyst while maintaining a suitable specific surface area, pore volume, and pore size, which are crucial for efficient catalytic performance.

**Table 2:** BET characterization of Ru–Sn/γ-Al_2_O_3_ catalysts with different Sn–Ru ratios

Sn/Ru (mol^−1^)	Specific surface area (m^−2^ g^−1^)	Total pore volume (cm^−3^ g^−1^)	Mean pore (size/nm)
0.2	203.78	0.406	7.9689
0.5	201.54	0.3731	7.4057
1.1	180.64	0.3751	8.3068

### Effect of reaction conditions on the catalyst

#### Effect of reaction temperature

**Fig. 11:**
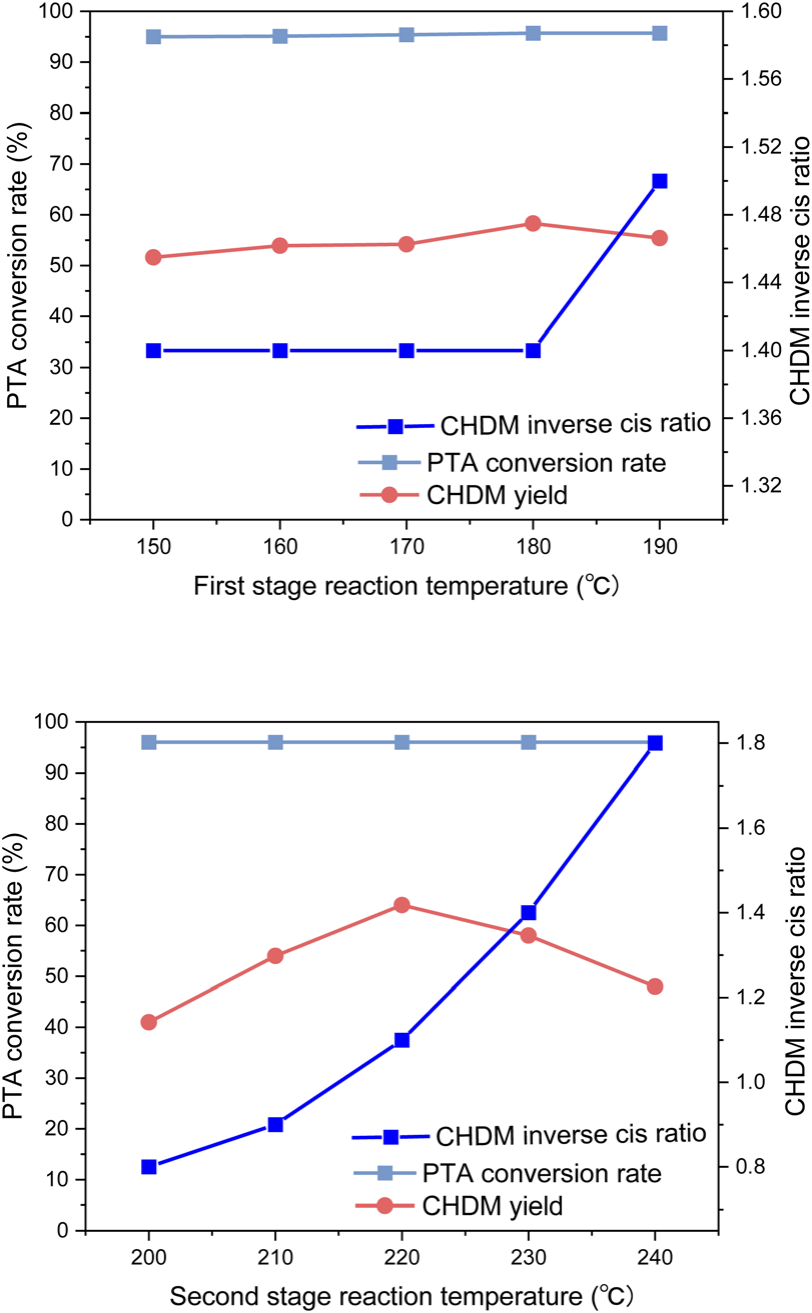
Effect of reaction temperature on hydrogenation reaction.

The reaction temperature is the key parameter of the hydrogenation reaction. As we all know, phenyl is easily hydrogenated to cyclohexane, while carboxyl is easily hydrogenated to aldehyde. To explore the influence of reaction temperature on the hydrogenation of PTA to CHDM, the optimum reaction temperatures for Pd/γ-Al_2_O_3_ and Ru–Sn/γ-Al_2_O_3_ catalysts were investigated as shown in Fig. 11. The reaction temperature strongly influenced the CHDM yield. A low temperature hindered the hydrogenation of carboxyl groups, while excessively high temperatures led to the breaking of C–C and C–O bonds, resulting in increased formation of by-products. Therefore, the reaction temperature in the second stage played a critical role.

It was found that the reaction temperature had little effect on the conversion rate, but mainly affected the yield of CHDM. In the first stage of the reaction, the reaction temperature has little effect on the yield of CHDM, and the optimal reaction temperature is 180 °C. In the second stage, which involves the hydrogenation of carboxyl groups, the reaction temperature strongly influenced the yield of CHDM. A low temperature hindered the hydrogenation of carboxyl groups, while excessively high temperatures led to the breaking of C–C and C–O bonds, resulting in the increased formation of by-products. Therefore, the reaction temperature in the second stage played a critical role. In addition, the reaction temperature in the second stage also affected the *anti-cis* ratio of CHDM. It was observed that the *anti-cis* ratio of CHDM increases with the increase of the reaction temperature in the second stage. This is due to the high internal energy and low thermal stability of *cis*-isomers. Therefore, the higher the reaction temperature, the higher the *trans* content of the target product CHDM.^35^ However, at 220 °C, although the yield of CHDM is the highest, the *anti-cis* ratio of CHDM is low. Considering the yield and *anti-cis* ratio of CHDM comprehensively, the optimal reaction temperature in the second stage is 230 °C. Overall, the experimental results indicated that achieving a high yield of CHDM requires careful consideration of both reaction temperature and pressure. While adjusting the reaction temperature alone might not be sufficient to achieve high yields, the influence of reaction pressure on the yield should also be taken into account.

#### Effect of reaction time

**Fig. 12:**
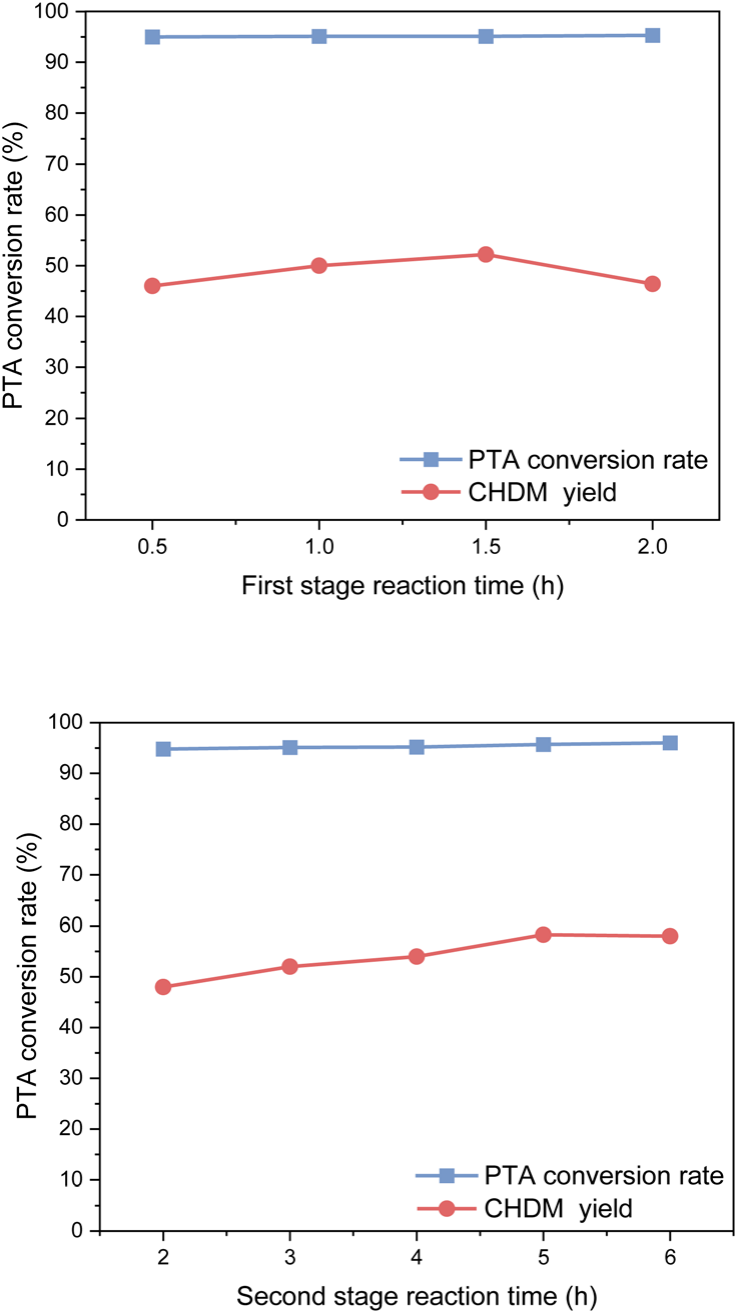
Effect of reaction time on hydrogenation reaction.

The effect of reaction time on PTA conversion and CHDM yield is shown in Fig. 12. From Fig. 11, the reaction time has little effect on the conversion rate of PTA because the hydrogenation reaction and hydrogenolysis reaction of PTA can occur simultaneously at high temperatures and high pressure. However, the reaction time played a crucial role in determining the yield of CHDM.

In the first stage of reaction, it was found that a reaction time was 0.5 h resulted in a yield of CHDM of 45.8%, whereas a reaction time of 1.5 h significantly increased the yield of CHDM to 52.2%. However, as the reaction time continued to increase, the yield of CHDM began to decline. This decline in yield can be attributed to the excessive hydrogenation of CHDM, leading to the formation of unwanted by-products. Consequently, the optimal reaction time in the first stage was determined to be 1.5 hours.

In the second stage, after 2 h of reaction time, PTA was completely reacted, and the yield of CHDM was only 48.2%. With the increase in reaction time, the yield of CHDM first increased and then decreased. This behavior can be attributed to the excessive hydrogenation of CHDM, leading to the formation of unwanted by-products. Thus, the optimal reaction time in the second stage was 5 h. It is worth noting that compared to the hydrogenation of the benzene ring, the hydrogenation of the carboxyl group to alcohol requires a longer reaction time. This indicates that the hydrogenation of the carboxyl group is a slower step in the overall reaction process.

#### Influence of reaction pressure

**Fig. 13:**
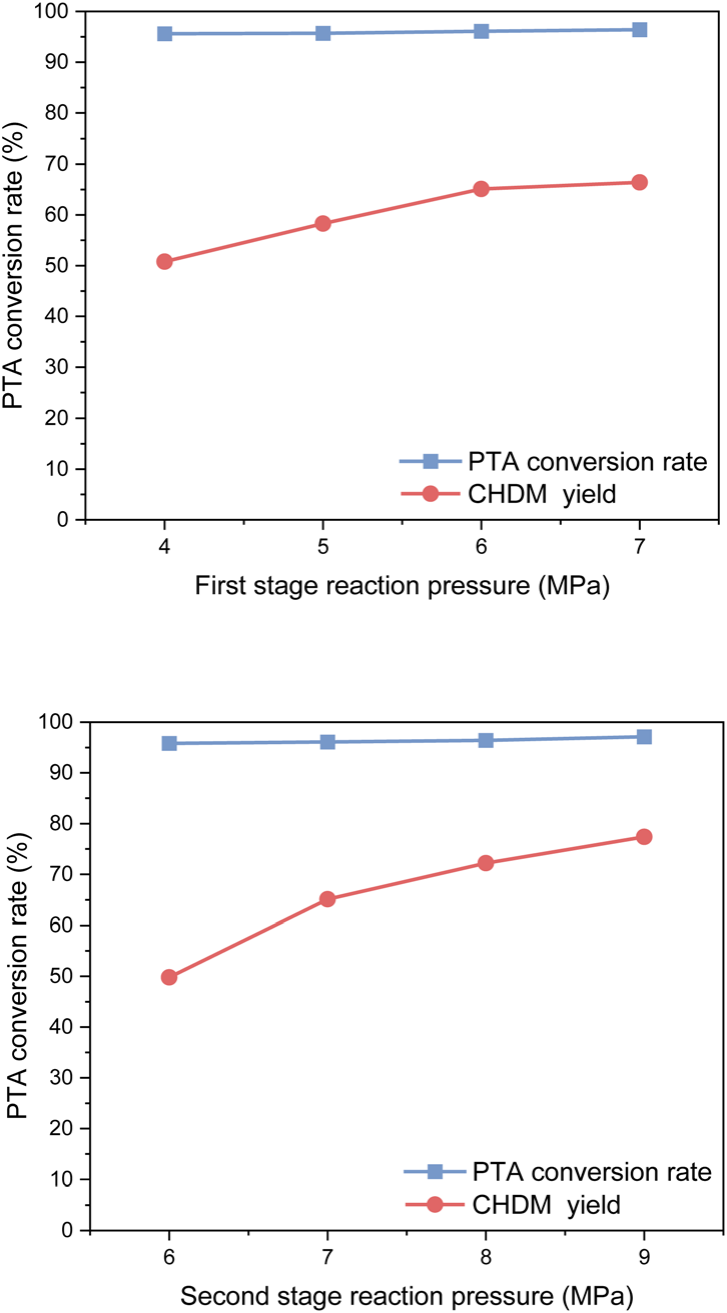
Effect of reaction pressure on hydrogenation reaction.

The influence of hydrogen pressure on PTA conversion is shown in Fig. 13. Hydrogen pressure plays a crucial role in the reaction, which can not only speed up the reaction but also promote the desired equilibrium shift. According to the experimental results, with the increase of reaction pressure, the yield of CHDM first increases and then tends to be flat. As the reaction pressure increased, the higher concentration of hydrogen in the system facilitated the hydrogenation reaction, leading to improved PTA conversion and increased CHDM yield. However, beyond a certain point, further pressure increases had a limited impact on the yield of CHDM.

Considering safety and economic benefits, an optimal reaction pressure of 6 MPa was selected for the first stage of the reaction, while 8 MPa was determined as the optimal reaction pressure for the second stage. These pressure values strike a balance between maximizing the yield of CHDM and ensuring operational safety and efficiency.

## Conclusions

A series of composite catalysts were prepared by the impregnation method for the hydrogenation of PTA to CHDM. The preparation of CHDM by one-step selective hydrogenation of PTA was realized, and it was found that Ru–Sn/γ-Al_2_O_3_ and Pd/γ-Al_2_O_3_ composite catalysts had a good performance in this process.

The results show that the Ru catalyst is active for the hydrogenation of C=C bonds while adding a small amount of Sn can make the catalyst activation for the hydrogenation of C=C and C=O bonds. The C=O hydrogenation rate of the Ru–Sn catalyst was significantly improved. The introduction of Pd is beneficial to the hydrogenation of the benzene ring and the increase of CHDM selectivity.

Using the three-metal composite catalyst with a Sn/Ru ratio of 0.5, the CHDM conversion of 96.3% and a yield of 72.2% were obtained. Moreover, the application of composite metal catalysts in the one-step hydrogenation method allowed for a reduction in the reaction pressure from 10 MPa to 8 MPa, making the process safer and easier to operate. Overall, the study demonstrates a promising approach for the one-step hydrogenation of PTA to CHDM, offering a new direction for catalyst research in this field.

## Conflicts of interest

There are no conflicts to declare.

## Supplementary Material
